# Isotope values of the bioavailable strontium in inland southwestern Sweden—A baseline for mobility studies

**DOI:** 10.1371/journal.pone.0204649

**Published:** 2018-10-04

**Authors:** Malou Blank, Karl-Göran Sjögren, Corina Knipper, Karin M. Frei, Jan Storå

**Affiliations:** 1 Department of Historical Studies, University of Gothenburg, Gothenburg, Sweden; 2 Curt-Engelhorn Center for Archaeometry gGmbH, Mannheim, Germany; 3 Environmental Archaeology and Materials Science, The National Museum of Denmark, Brede, Denmark; 4 Department of Archaeology and Classical studies, Stockholm University, Stockholm, Sweden; University at Buffalo - The State University of New York, UNITED STATES

## Abstract

The inland area of southwestern Sweden is well known for its well-preserved archaeological animal and human remains dating back to the Mesolithic and Neolithic (10000–4000 and 4000–1700 BC). They allow application of multiple bioarchaeological methods, giving insights into various and complementary aspects of prehistoric human life, as well as economic and social structures. One important aspect concerns human mobility and its relation to social networks and to circulation of objects. Here, strontium isotope analysis plays a crucial role. The present study aims to construct a strontium isotope baseline of southwestern Sweden with considerably greater coverage and higher resolution than previously published data. As the region has been affected by glacial events, the relation between bedrock geology and isotope signals of the bioavailable strontium in such areas is given special attention. We determined strontium isotope ratios for 61 water and five archaeological animal samples, and combined the data with previous measurements of two water and 21 non-domestic faunal samples. The results reveal a complex pattern. Several areas with distinct baseline ranges can be distinguished, although with overlaps between some of them. Overall, the bioavailable strontium isotope signals mirror the basement geology of the region. The highest ratios occur in the geologically oldest eastern parts of the Precambrian terrain, while lower ratios are found in the western part, and the lowest ratios occur in the youngest Paleozoic areas. At the same time, there are minor deviations compared to the underlying bedrock, due to glacial transport, overlying sediments, and local intrusions of younger rocks. The background data set now available allows for more nuanced and detailed interpretations of human and animal mobility in the region, in particular by identification of subregions with differing strontium isotope ratios within the Precambrian province. Also, we can now identify long distance mobility with greater confidence.

## Introduction

This study aims to construct a strontium (Sr) isotope baseline of southwestern Sweden to provide a foundation for archaeological studies of human and animal mobility. In general the preservation of bones and teeth in Sweden is poor due to the acidic soils. However, there are areas with favourable geologic conditions for bone preservation, including the sedimentary regions of southwestern Sweden with calcareous soils. Here, human and animal bones dating back to the Mesolithic (10000 to 4000 cal BC) have been found, but the Neolithic (4000 to 1700 cal BC) and later periods are also well represented.

**Fig 1:**
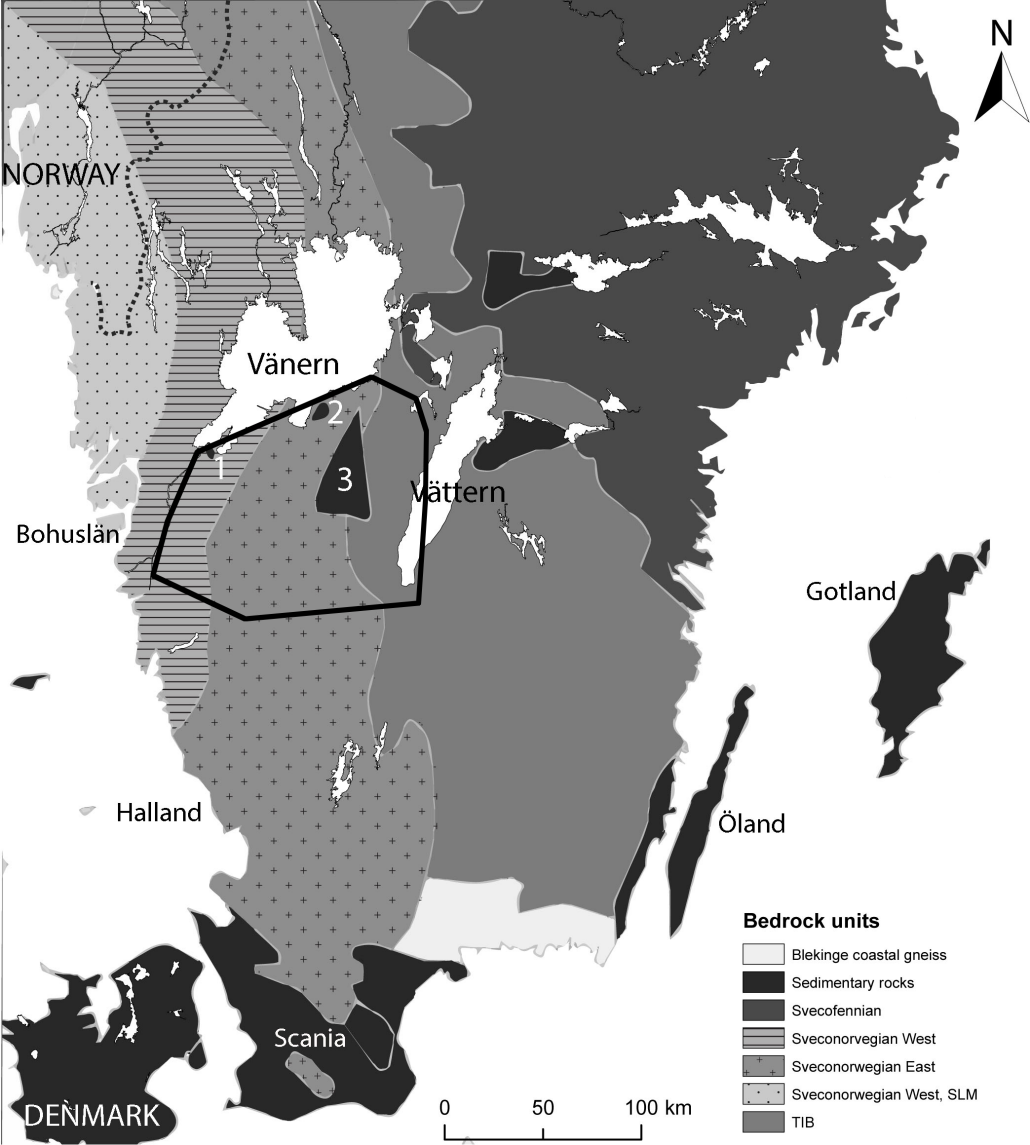
Simplified bedrock geology of southern Sweden, by Malou Blank. Based on geological maps from the Geological Survey of Sweden, data (Berggrund 1:1 million). Background map created using data from Esri. Data and maps licensed to University of Gothenburg. 1: Halle and Hunne mountains, 2: Kinnekulle, 3: Falbygden. Investigated area marked with black line.

In the sedimentary area of Falbygden (Fig 1), one of Northern Europe’s largest concentrations of early Middle Neolithic (3300 to 2800 cal BC) passage graves and Late Neolithic (2350 to 1700 cal BC) gallery graves occurs, producing a remarkable assemblage of well-preserved animal and human remains [1–6]. This also applies to later periods, such as for instance the abundant assembly of skeletons from Varnhem, one of the earliest Christian cemeteries, dating back to 900 cal AD [7]. The clear spatial structure of the geology and the well preserved human and animal bone material makes this area an unusually fruitful case study for investigations combining bioarchaeological and archaeological data in order to understand prehistoric economy and society.

Since Falbygden is essentially an”island” of Cambro-Silurian sedimentary rocks surrounded by older Precambrian crystalline rocks, it constitutes an ideal setting for mobility studies by way of isotopic analysis. A clear distinction between the Cambro-Silurian and the Precambrian regions was indicated by previous Sr isotope studies [8, 9]. However, these studies leave several issues regarding the background isotope variation unresolved, due to a rather low number of sample sites. This concerns for instance the variation between and within different Precambrian provinces, the possibility of spatial variation within the Cambro-Silurian region, and the effect of glacial transport of sedimentary rocks into the Precambrian area to the south of Falbygden.

This baseline study is part of ongoing research on Middle and Late Neolithic human and animal mobility in western Sweden. Regional networks and contacts with southern Scandinavia and indirectly with continental Europe during the Neolithic can be traced by building traditions, burial practices and imported goods [10–13]. The Middle Neolithic remains have been focus for a number of scientific analyses, including osteology, aDNA, ^14^C dating, stable isotopes, Sr and sulphur isotopes, etc. [1, 3, 6, 8, 9, 12, 14–17]. Through these studies, the population in Falbygden has been shown to be part of a much larger system of exchange/alliances with surrounding Precambrian regions, involving humans but in particular also cattle. Furthermore, Sr isotope results have indicated an increased human mobility and an increased variation in the isotope ratios during the Late Neolithic [18, 19].

Previous studies suggested that ca 25% of the people buried in Falbygden passage graves originated from areas with Precambrian bedrock, and the variation in the in the Sr isotope signals suggest that several different regions were involved. For cattle, the number of imported animals was even higher (>50%) and the Sr isotope signals indicate more varied and partly different areas of origin from those of humans. At the same time, clear cases of long distance movement seem exceedingly rare, in some contrast to the archaeological evidence. This raises questions about the limitations of mobility studies by isotopic analysis, but also highlights the limitations of our present knowledge about Sr isotope variation in Scandinavia.

In order to better understand the functioning of these complex exchanges, we need a better grasp of Sr isotope variation over a larger area, particularly in the Precambrian provinces. This is a prerequisite for more elaborate discussions of different kinds of mobility which still are not addressed sufficiently [20–23]. In combination with other archaeological and bioarchaeological data, this would involve issues such as the temporal and spatial scale of individual movement, different movement patterns for specific categories of people, the mobility patterns of people versus those of animals, and possible social drivers behind these patterns. Such issues will be discussed in upcoming papers, evaluating isotope data from humans and domestic animals against the background variation presented here.

The necessary level of detail of the background data is dependent on the underlying geological complexity, as well as on the research questions asked, and no general rules can be formulated. Numerous environmental samples from archaeological sites as well as from the surrounding areas are needed not only to establish the local bioavailable range but also to consider or rule out potential areas of origin of non-local individuals. A baseline of southwestern Sweden is also important for mobility studies in neighbouring regions. Archaeological human and animal materials from southern Sweden are at present included in several mobility research projects (e.g. [24–27]).

Baselines have previously been produced for e.g. the British Isles [28, 29], France [30], Denmark [31–33], the Netherlands [34], Switzerland [35], parts of the North Atlantic regions [36] and in parts of Mesoamerica [37].

In Sweden, background data are only available for a few restricted areas mostly in connection to specific archaeological sites [8, 9, 19, 24, 38–48]. An overview of Sr isotope ratios in southern Sweden has been published by Blank and Knipper [19]. In addition, there is an ongoing study investigating the Sr isotope baseline of the west coast of southern Sweden [25, 26] which will be an important complement to this study.

In order to expand the knowledge of Sr isotope spatial variation in western Sweden, we measured a total of 61 water and five animal samples from an area covering 120 x 130 kilometres. These were combined with previous data from two water samples and 21 samples from non-domestic small fauna, to obtain as high resolution as possible (S1 Appendix).

## Background

### Strontium isotopes and mobility

Strontium isotope analysis has become an important method in provenance studies in a wide range of fields, including archaeology [49– 51], ecology [52], food [53–55] and forensic science [56, 57]. In archaeological research, Sr isotope analysis was introduced by the pioneering work of Ericson [50] and has become a standard method for studying human and animal mobility. In many cases, this method has given us insights into prehistoric socio-dynamics that would not be possible with other methods [9, 18, 58–69].

Strontium is an alkaline earth element which has four naturally occurring isotopes; three stable and one, ^87^Sr, radiogenic. The ratio between two of them, ^87^Sr/^86^Sr, is used in provenance studies. The radiogenic ^87^Sr is formed by radioactive β-decay of ^87^Rubidium [70]. Different rocks are therefore characterised by ^87^Sr/^86^Sr ratios dependent on the original content of rubidium as well as on the formation age [70, 71]. Bioavailable Sr originates mainly from weathering rock minerals. However, weathering is a process that affects different types of minerals unequally, which in themselves vary in both Sr content and Sr isotope ratios. Bioavailable Sr ranges may therefore vary from those of the parent bedrock as they represent a mixture of Sr ratios from different sources [72, 73]. The element passes from soils and water into the biosphere and food chain and further into the human skeleton with minimal biological isotope fractionation [49, 74, 75]. Hence, the ^87^Sr/^86^Sr ratio is effective for identifying human and animal mobility. It is assumed that human and animal Sr isotope signals give a weighted average of the bioavailable ^87^Sr/^86^Sr ratios within their home range [49]. Comparing isotope values in for example human tooth enamel, which is formed during childhood, to those of samples that reflect the bioavailable Sr around the archaeological site can thus identify possible movements of individuals and mobility patterns of populations.

In addition to weathering rocks, there may also be contributions from precipitation, windblown dust, fertilizers, and sea spray [49, 51]. The impact of such factors has been discussed, but may in many cases be considered not to be large enough to obscure the signal from the local geology. According to Frei and Frei ([32]:157), rainwater does not have measurable impact on the Sr isotope composition of surface water in Denmark. These authors argued that this might be due to the fact that soils in Denmark are partially composed of Sr rich limestone derived material, which are the dominant Sr source in surface water and the low Sr concentration in rainwater. The potential impact of Sr from modern sources, such as fertilizers, to the local Sr isotope baseline ranges has also been debated [31, 49, 76]. Frei and Frei [31] argued that only extremely high amounts of fertilizers that are currently used within the Danish regions would alter the Sr isotope signals of the Danish soils and surface waters. Nevertheless, to avoid the influence of fertilizers as much as possible, samples should be taken away from modern agricultural areas. Wind transport of airborne sea salt in southern Sweden was studied by Gustafsson and Franzén [77] in a 300 km transect. They found that sea salt was transported all over the transect, but with strongly decreasing concentration after ca 20–30 km from the coast, after which concentrations stabilized on a very low level.

Tooth enamel is the preferred material for investigating prehistoric human and animal mobility, as it is less susceptible to diagenesis and contamination than bone [49]. Bone undergoes continuous chemical and structural turnover, while enamel stays stable after the formation which in humans takes place in infancy to early adolescence, depending on tooth type [78]. In case a person moves to a new location with a different geology after or during the tooth enamel formation, the Sr isotopic ratio of the enamel will differ from the isotopic signal of the new location, under the premise that local food sources were consumed. Seasonal mobility or movement during the time of tooth formation may result in mixed isotope ratios, as tooth crystallization sometimes can take several years [78]. Teeth with enamel formed at different stages and micro sampling from different levels of the tooth crown (mostly conducted on animal teeth) can give us more detailed information about the movements [61, 79, 80].

### Sample material considerations

In previous Sr isotope research the terms baseline and isoscape have been used variedly (e.g. [34, 81]). Here, we use these terms as equivalent, referring to the background variation of bioavailable Sr isotope ratios within a region. It is implied that isotopic variation is inherently spatial, and that it is the systematic spatial variation over a region that is of interest, rather than the variation at any specific point, such as a find site. A baseline should not be considered as fixed or stable, as new data accumulate over time, adding more nuances to the picture.

The uptake of Sr in humans and animals is mainly controlled by Sr in plants, animals and drinking water coming from a certain area, or from a number of areas visited during a period of time. As discussed above, the Sr isotope ratios characteristic of these different sources can vary and so do the Sr concentrations ([82]: 187). Strontium concentrations are generally higher in plants than in meat, and some researchers argue that most Sr in humans originates from plants while the contribution from animals is of less importance [49]. Frei and Frei [31] also suggest drinking water as a potentially important source of Sr in humans, and recent investigations on modern humans show that local water may be an important source of Sr to human hair [83]. In a controlled feeding study on pigs, Lewis et al [75] found the contribution from drinking water to be less than 5%, however, even with a rather high Sr concentration (2.6 ppm) in the water. The Sr concentration in surface water varies depending on the local geology. Generally, Sr concentrations in surface waters are higher in areas dominated by calcareous rocks (eg. limestones), whereas they are lower in areas dominated by mafic rocks and granitoids. In southwestern Sweden, the Sr concentrations measured by us are very low (Sample preparation and analysis). Therefore, water should be expected to contribute very little to human Sr uptake in our study area. Regardless of the contribution of water to human and animal values, however, such samples are still useful as a proxy for bioavailable Sr isotope ratios [31, 49].

There is at present little consensus on what proxy to use, and the choice of proxy material has been debated [28, 31, 49, 72, 76, 82, 84]. Ongoing methodological development, economical resources, availability and accessibility are some important factors in this choice. Various materials have been suggested including archaeological fauna, modern fauna and flora, human bone, soils and water. Often, isotope studies include several different sample types. Grimstead, Nugent and Whipple [82] advocate a broad sampling strategy of soil, archaeological fauna, vegetation and water to map the Sr isotope system. However, these authors recognize and encourage that current provenance investigations have shifted away from utilizing animals to create baselines [82].

Low mobility faunal species have often been suggested as good reference samples [8, 33, 85]. It is common to use enamel and bone from small mammals and shell from snails, as these animals are unlikely to move over large areas [8, 33, 36, 45, 47, 48]. In some of these studies, both archaeological and modern animals were included. In modern samples, the consumption of non-local foodstuffs cannot be excluded off-hand, and this could also be the case for archaeological domestic animals ([49]: 158). In addition, although the home range of rodents is small, they may sometimes have moved or been transported over large distances both in modern and prehistoric contexts [82, 86]. In a few studies, the Sr isotope signal in snail shells appears to be shifted towards that of rainwater, 0.709 ([28]: 3, [76]: 225).

Domestic species, including dogs, cattle and pigs, have also been used for establishing the local Sr isotope signal [36, 47, 66]. However, using these animals as proxies is problematic as we need to consider the possibility of circulation of domestic animals in social networks as well as of seasonal movements of the animals [9, 58, 61, 62, 87]. Human samples are also referred to [36, 45]. In these cases, one needs to be careful not to end up in a circular reasoning, in that the target materials for which the origin is sought is used to define the local baseline. Evans et al. ([28]: 3) used data from diagenetically altered bones, as Sr in groundwater from the burial site was most likely incorporated into these bones. Hence, the Sr signal of the bones would reflect the local Sr signal of the site.

Another suggested proxy is modern soil and plant samples [24, 30, 36]. Soil samples capture microvariation, which may not be relevant for humans and other larger animals, who average the Sr intake over their home ranges. When soil is used, the depth of the sample needs to be considered, as the Sr isotope ratio might vary with depth ([31]: 337, [49]: 151, [88]: 316, [89]). Moreover, depending on the leaching technique used during sample preparation, the same soil sample may produce different ^87^Sr/^86^Sr ratios, of which not all reflect the bio-available fraction. The topsoil also seems to retain the Sr isotope ratio of rainwater [28]. Likewise, plants from the same location with different root depth can have different isotope signals. Atmospheric factors such as windblown dust may also affect plant samples [49, 82]. Frei and Frei ([31]: 338) conclude that the Sr isotope signal of topsoil and small herbivores in Denmark was 0.15% lower than that defined by lake and stream water, and suggest that this might be due to rainwater and soil leaching/weathering of radiogenic components of Sr in the soil.

According to several studies [28– 32, 76], water appears to be a good proxy for identifying the isotope signals relevant to humans. In a study conducted by Maurer et al. [76] Sr isotope ratios in different biological and geological samples were compared, and the authors concluded that the best reference samples with the most accurate estimates for the bioavailable signal were water and tree leaves ([76]: 227). In a study conducted by Wickman and Åberg [90], water samples from rivers in northern Sweden and Finland seemed to correlate rather well with the values from the local bedrock. The published baselines of the British Isles [28] and of Denmark [31, 32] are based on numerous samples from surface water. Also, according to Bentley ([49]: 144), samples from rivers and streams are a good basis for the local isotopic ratio, as the composition of various eroded rocks represents the Sr available for plants and animals. Frei and Frei ([31]: 328) argue in their study that the Sr isotope signal of animal hair (sheep) mirrors the signal of surface water, and proposed that water is a good proxy to delineate the local baseline.

To sum up, there are a number of different proxies which are currently used to construct Sr isotope baselines. Considering that this study first and foremost was intended for archaeological research, focusing on humans and domestic animals, water was concluded to be the most suitable proxy and was used as the main source for this investigation. An important factor in this choice was accessibility and availability, as archaeological faunal remains are generally not preserved in the Precambrian area, while numerous springs and small streams are found all over the region. We also wished to avoid Sr from modern and non-local fauna, and we therefore minimized the samples from animals and only use enamel values from small, non-domestic fauna. Further, soil and plant samples were avoided, soil because the relevance for bioavailable Sr ratios is uncertain, and plants because of possible contamination problems from wind-blown dust.

### The geology of southwestern Sweden

The oldest bedrock formation in Europe is the Baltic shield which extends over the Scandinavian Peninsula, Finland and parts of Russia. Within this formation, the oldest, Archaean rocks, which are 3.4 Ga (billion years old), occur in western Russia, eastern Finland and Karelia. As the shield has grown in a westerly direction, the western parts are younger [91, 92]. The Baltic shield is divided into several provinces, including the Svecofennian province (1.9–1.75 Ga) comprising parts of eastern Sweden and Finland, the Sveconorwegian province (1.7–0.9 Ga) covering western Sweden and Norway, and the Transscandinavian granite-porphyry belt (TIB, 1.8–1.6 Ga) which extends between these two provinces (Fig 1). The Sveconorwegian province is usually divided into an eastern and a western segment and was subject to extensive metamorphism [93].

The Baltic shield is made up of Precambrian gneisses and granites [91]. In geological research large numbers of ^87^Sr/^86^Sr determinations on whole rock samples from southern Scandinavia have been conducted for dating purposes (http://maps.sgu.se/sguinternetmaps/alder/viewer.htm). ^87^Sr/^86^Sr ratios of Precambrian crystalline rocks are elevated (radiogenic) with ^87^Sr/^86^Sr > 0.722 ([8]: 89). Gneisses have more heterogeneous Sr isotope signatures than their unmetamorphosed counterparts, the granites [8]. In the Svecofennian province in eastern and northeastern Sweden, Sr isotope values are generally higher than 0.73 [88, 91, 92, 94, 95].

**Fig 2:**
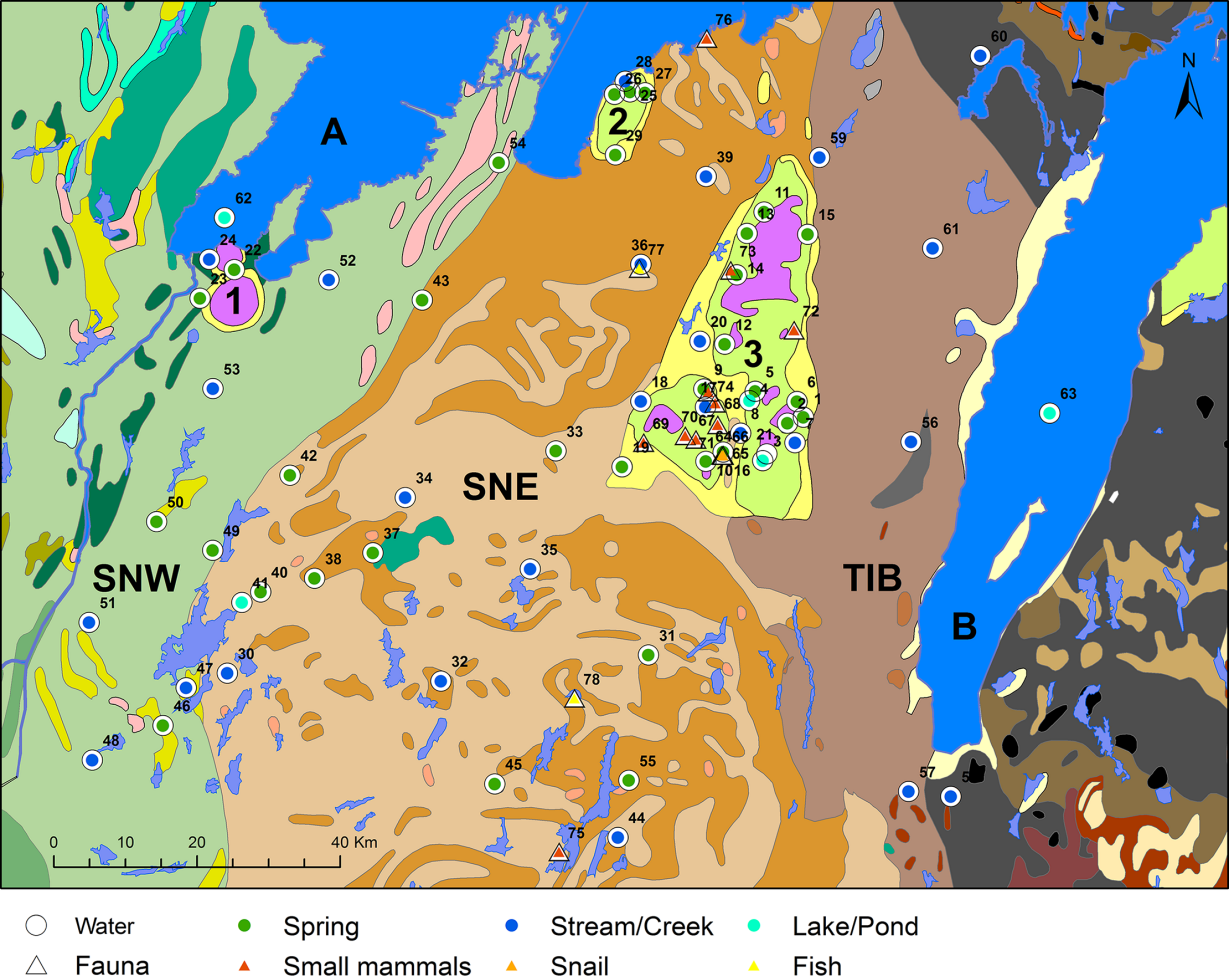
Sample locations projected on a geological map of southwestern Sweden, by Malou Blank. Data from the Geological Survey of Sweden (Berggrund 1:50000). Background map created using data from Esri. Data and maps licensed to the University of Gothenburg. For sites numbers see S1 Appendix. 1: Halle and Hunne mountains, 2: Kinnekulle, 3: Falbygden. SNW: Sveconorwegian west, SNE: Sveconorwegian east and TIB: Transscandinavian granite-porphyry belt. A: Lake Vänern, B: Lake Vättern. Colours of the subunits are explained in S2 Appendix.

Most of southwestern Sweden consists of Precambrian crystalline rocks, from the Early and Middle Proterozoic eras ([93, 96] S2). The area, also known as the west Swedish Gneiss Province, is subdivided into an eastern and a western segment. As shown in Fig 2, the bedrock geology has been further divided into subunits, with different mineral and chemical composition, which are described in S2 Appendix.

In addition, there are also pockets dominated by younger rocks with different formation histories. There are three areas characterized by Paleozoic sedimentary rocks, Falbygden, Kinnekulle and the Halle/Hunneberg mountains. These rocks once covered large areas, of which now only some areas remain due to dolerite/diabase caps protecting them from the glacial ice. Devonian to Permian dolerites, 350 to 250 Ma (million years) in age, intruded at different levels into the sedimentary succession [97].

Covering an area of 50 x 30 km, Falbygden is the largest of these sedimentary terrains (No. 3, Fig 2). Here, a series of sedimentary rocks were deposited in the Lower Paleozoic about 550 to 400 Ma ago. The sedimentary successions include Cambrian, Ordovician and Silurian alum shale, limestone, and slate, on top of sandstone deposited during the lower Cambrian. These rocks are partly covered by dolerite, which now forms the tops of table mountains. A similar remnant of Cambro-Silurian sediments occurs at Kinnekulle (No. 2, Fig 2) near Lake Vänern, 30 km north of Falbygden. At the southern tip of Lake Vänern another very limited area with lower Cambrian sandstone overlaid by Cambro-Silurian sedimentary rocks occurs, the Halle and Hunne mountains. Also here, the sedimentary sequence is covered by Upper Paleozoic dolerite (No. 1, Fig 2, [96]).

Finally, local occurrences of intrusive igneous rocks such as gabbro are found within the Precambrian terrain. Normally, these are of limited extent and their influence on Sr isotopic ratios is expected to be marginal or very localised.

The geology of western Sweden is complicated by metamorphic overprinting and much younger glacial erosion and deposition of glaciogenic material which today shapes the landscape. The bedrock is in most areas covered by glacial deposits (moraines, glaciofluvial deposits), marine, and by ice lake, and freshwater lake sediments of variable thickness. These deposits add to the complexity of bioavailable Sr and may affect or mask the signatures characteristic of the bedrock.

Moraine is by far the most common coverage in the region. Moraine consists of a mixture of materials partly different from the local bedrock and material may be deposited at a distance of several hundred km from where they originally eroded, although the distances are normally in the order of a couple of km [98–100]. In the study area, the ice sheets moved in a south-westerly direction [101, 102]. The effects of glacial processes on the Sr isotope ratios are a smoothening of small-scale local variations and a geographic skewing in the direction of ice movement [100]. Gillberg [101] studied the composition of moraines in the present study region, and showed that limestone was present at levels >1% in the area to the south of Falbygden, at a distance of up to 20 km from the limestone edge. Closer to the edge, the limestone content was gradually higher. A similar gradient has been observed in the CaCO3 content of the moraine ([103]: 65).

In depressions, late glacial ice lake and marine as well as postglacial lake sediments have been deposited on top of the moraine. The material in these sediments largely consists of redeposited moraine material, predominantly composed of eroded Precambrian basement rocks. Marine sediments are today found below the marine limit, ca 130 masl in the region of Falbygden. Ice lake sediments are found mainly in basins and depressions to the east of Falbygden, while lake sediments are found in low lying areas of the Väner basin [91, 102]. As Falbygden is situated above the marine limit, no sediments are found here, and the area is covered by moraine.

## Method and material

### Sample material

The study area is rich in natural springs, small streams and lakes which are suitable for water sampling, and have most probably been important sources for drinking water both for humans and animals in prehistory. The samples in this study consist of ground water from springs and surface water from creeks, streams and lakes (Table 1). The different sources have different uptake areas, thus reflecting different Sr sources. Springs reflect the Sr of various lithological layers and can also be influenced by surface water. Streams and creeks have different and often larger catchment areas and percolate through different lithologies and the Sr isotope ratio is a mix of various water sources with addition of rainwater and soil signals. As the studied area has not been affected by any major environmental changes since prehistory we assume that the bioavailable Sr measured in the modern samples reflects prehistoric conditions.

**Table 1:** Number and type of samples.

Sample type		This study	Previous studies	Total
Water	Spring	32	0	32
	Creek, stream	25	0	25
	Lake, pond	4	1	5
	Unknown	0	1	1
Fauna	Enamel	5	17	22
	Bone	0	1	1
	Shell	0	3	3
Total		66	23	89

A total of 61 water samples were collected for this study. Two previously published results from Lake Vättern and Hjortmossen were also included [41, 90]. In addition, enamel from five small non-domestic mammals was sampled from archaeological sites in Falbygden (S1 Appendix). Furthermore, 21 enamel and shell samples of low mobility animals (18 small mammals and three snails) were included from previous studies [8, 9]. The snail samples were modern, and even though this species tends to show lower ^87^Sr/^86^Sr values than the Sr in other species, it still reflects the bioavailable Sr. The mammals were found in archaeological contexts, but a modern/historic origin cannot be ruled out. These values were supplemented by measurements from fishes (three teeth and one throat bone) which were consistent with other local Sr isotope ratios ([8], S1 Appendix). The purpose of the selected animal samples was to complement the water samples and to investigate if the different sample types coincided or not. Some of the samples from the previous study [8] were excluded as they originated from larger mammals with larger home ranges. Table 1 lists a summary of samples used herein. Ethical statements are not relevant to this study. No specific permissions were required for the collection of water samples, according to Swedish legislation. Permissions from the museum of Västergötland and the Swedish History Museum were granted to conduct destructive analysis on the archaeological animal samples.

### Sample collection

Water samples were collected between October 2014 and December 2016. Sampling was avoided in connection with heavy rainfall and ice melting as water Sr isotopic values can change during these periods [51, 90]. This investigation covers a geographical area of 120 x 130 km with the highest concentration of samples in Falbygden (Figs 1 and 2). Our sampling strategy was aimed at achieving a reasonable geographical spread while covering all the different lithologies present in the area. In addition, the proximity to archaeological sites, as well as the accessibility of suitable water was considered. Water samples were collected from 20 localities in the sedimentary area of Falbygden, from 8 localities in the sedimentary area of Kinnekulle, Halle and Hunne mountains, from 32 localities in the Precambrian areas, plus one sample from the Lake Vänern (Fig 2, S1 Appendix).

Five small mammals from archaeological sites in Falbygden were sampled with the help of Maria Vretemark at Västergötlands Museum at two different occasions (Fig 2, S1 Appendix). These samples were not ^14^C dated, however their contexts indicate that they most likely are prehistoric (S1 Appendix). The sampling and preparation process of previous fauna materials is described in Sjögren, Price and Ahlström [8]. Details of the preparation and analysis of the additional two water samples are available in Frei et al. [41] and Wickman and Åberg [90]. In Fig 2 all sample locations are plotted.

### Sample preparation and analysis

Water and animal enamel sample preparation and analysis were carried out at the Curt-Engelhorn-Center for Archaeometry gGmbH, Mannheim, Germany. The samples were centrifuged and filtered in cases of high particle loads. 10–20 ml of water were dried down and the residue taken up with 250 μl of 3 N HNO_3_. Sr separation was carried out in Teflon columns with Eichrom Sr-Spec resin. The columns were preconditioned with 500 μl of 3 N HNO_3_, the samples loaded and washed in with 3 x 400 μl of 3 N HNO_3_. The Sr was eluted with 1.5 ml of 0.4 N HNO_3_ (0.5 ml + 1 ml steps). In order to provide always sample solutions with the same Sr concentration for the High-Resolution Multi Collector-ICP-MS (HR-MC-ICP MS, Neptune), we determined the Sr concentrations after the column chemistry using a Quadrupole-Inductively Coupled Plasma-Mass Spectrometry (Q-ICP-MS). Strontium concentrations of the water samples ranged between about 5 and 150 ppb. Using 20 ml of water from each sample ensured to elute enough Sr for isotope ratio determination, based on 1 ml with 100 ppb of Sr in three blocks of 30 lines each. Raw data were corrected according to the exponential mass fractionation law to ^88^Sr/^86^Sr = 8.375209. Blank values were lower than 10 pg Sr during the whole clean lab procedure.

Small mammal teeth were washed in distilled water and enamel samples separated from adhering dentin. Because of the small amounts of samples, no pretreatment with acetic acid was performed. About 3–5 mg of enamel were dissolved in 250 μl of 3 N HNO_3_ and the Sr separated using Sr-Spec resin as described above for the water samples [63, 64, 104]. During the course of the analyses, the Eimer and Amend (E & A) standards yielded ^87^Sr/^86^Sr ratios of 0.70803 ± 0.00004, 2 SD; n = 63 [inter-laboratory mean: 0.70827 ± 0.00004 1 SD [105]; in-house long-term average Jan. 2011-June 2018: 0.70801 ± 0.00008 2 SD (n = 1240], and the NBS 987 standard yielded 0.71025 ± 0.00004, 2 SD; n = 55 [certified value: 0.71034 ± 0.00026 (95% confidence interval)]. Standards and samples were measured in the same concentrations of 100 ppb Sr.

### Statistical methods

Statistical analyses were performed using IBM SPSS version 23. The data evaluation included descriptive statistics and significance tests. As a normal distribution could not be demonstrated in all cases, the non-parametric Mann-Whitney U-test (MWU-test) was used to examine whether the observed differences were statistically significant at the 5% level. This test was preferred because of the sometimes small sample sizes and differences in sample sizes between groups. The Mann-Whitney U-test was used to compare distributions across groups based on sample type, bedrock geology and location.

**Fig 3:**
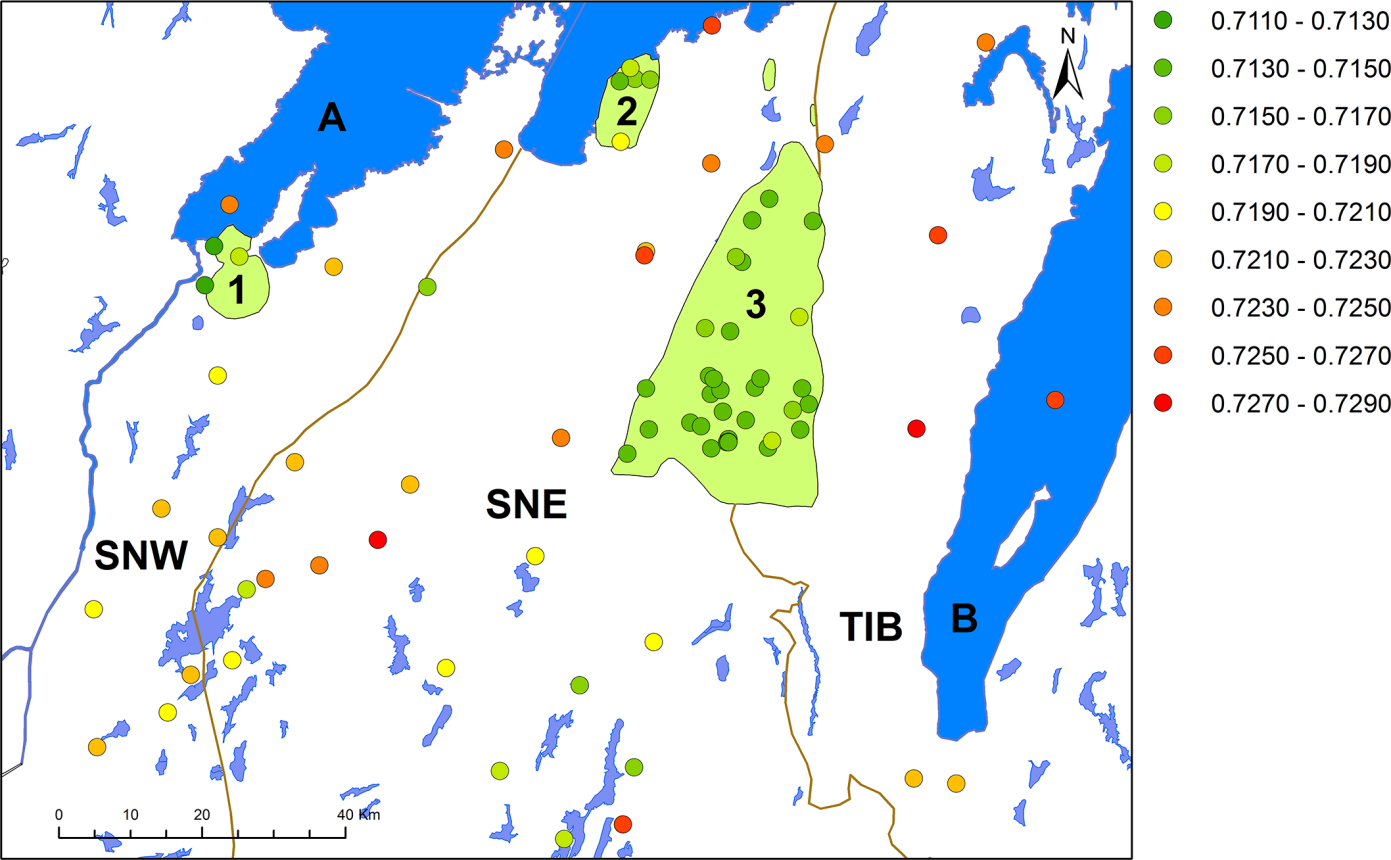
Strontium isotope ratios at the sampled locations, grouped at intervals of 0.002 into 9 classes, by Malou Blank. Background map created using data from Esri. Data and maps licensed to the University of Gothenburg. 1: Halle and Hunne mountains, 2: Kinnekulle, 3: Falbygden. A: Lake Vänern, B: Lake Vättern. SNW: Sveconorwegian west, SNE: Sveconorwegian east and TIB: Transscandinavian granite-porphyry belt.

ArcGis 10.1 was used to plot the isotopic values at the sampled locations, using various methods of classifications and numbers of classes depending on scale (Figs 3 and 8). ArcGis was also used to interpolate the Sr isotopic ranges in the studied area. An interpolated surface was produced by a standard circular Empirical Bayesian Kriging method. The Empirical Bayesian Kriging is an interpolation method that accounts for the error in estimating the underlying semivariogram through repeated simulations. This method permits accurate predictions of moderately non-stationary data and is more precise than other kriging methods for small datasets [106]. In this case the method was based on 100 simulations with a searching neighborhood of four sectors and a maximum of 15 and minimum of three neighbors.

## Results and discussion

The Sr isotope ratios of all samples are presented together with lat/long coordinates (WGS 84), context, and geological information in S1 Appendix. For samples collected from archaeological sites, site number in the national Sites and Monuments register are also included. Faunal samples from previous studies, excluded from the present study are coloured red.

Initially, different sample types were compared for areas where there is close spatial overlap (S2 Appendix). No statistically significant differences between the two categories of water or between the water and fauna samples were observed (Tables A, B, C and Fig C in S2 Appendix). Hence, we consider the fauna (low mobility animals) and water (ground and surface water) samples to be compatible and our two data sets were combined to achieve the best resolution possible.

### Strontium isotope ratios and bedrock geology

**Fig 4:**
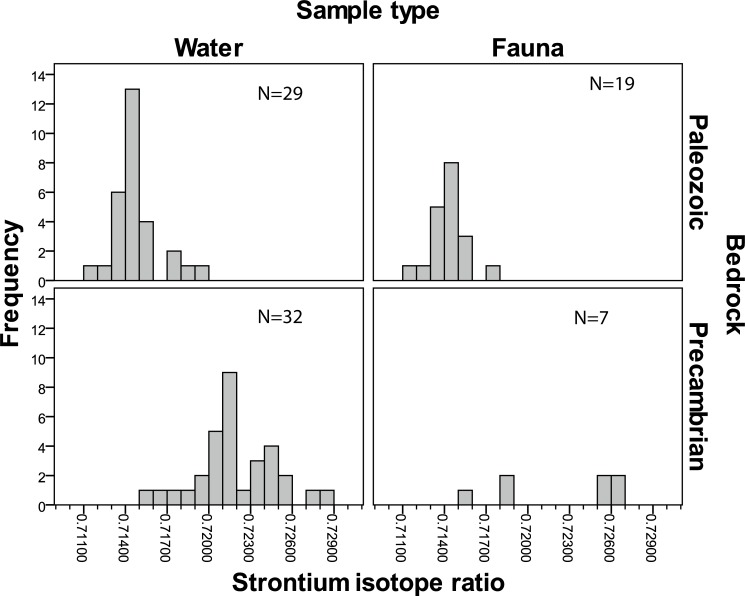
Histogram of Sr isotope ratios of water and animal samples from the Paleozoic and the Precambrian areas.

In Fig 3, the Sr isotope values are plotted for all samples, and a histogram of Sr isotope ratios by bedrock type is given in Fig 4. The general trends are clearly visible: lower isotope values in the Paleozoic areas of Halle and Hunne mountains, Kinnekulle and Falbygden, and higher values in the Precambrian terrain (Figs 3 and 4). This confirms the results from earlier studies. The isotope ratios in the Precambrian regions are internally varied with higher ratios in the east (Fig 3). These trends will be investigated more in detail by statistical testing. First, data from the older (Precambrian) areas are compared with the values from younger (Paleozoic) rocks. Thereafter, we discuss data from the Precambrian areas in more detail. Last, we analyse data from the Paleozoic sedimentary and intrusive regions.

#### Precambrian and Paleozoic bedrock

In Table 2 statistics of Sr isotope values of samples from Precambrian, Lower Paleozoic and Upper Paleozoic areas are summarized. The Precambrian terrain in the study area stretches from the Sveconorwegian west (SNW) and the Sveconorwegian east (SNE) segments to the TIB, while the Paleozoic regions are located in Falbygden, in the Kinnekulle and the Halle and Hunne mountains (Fig 2). The mean and median Sr isotope values are highest in the Precambrian regions and lowest on the Upper Paleozoic formations. The small sample size of the Upper Paleozoic group is addressed in the section Paleozoic area.

**Table 2:** Summary statistics of Sr isotope ratios of water and animal samples by formation age.

Formation Era/period	Bedrock	Age of formation	N	Mean	Std. Dev.	Median	Min.	Max.
Upper Paleozoic, Devonian- Permian	Intrusive: diabase	350–250 Ma	3	0.71383	0.00297	0.71243	0.71181	0.71724
Lower Paleozoic, Cambro- Silurian	Sedimentary: slate, limestone, alum shale and sandstone	550–400 Ma	45	0.71463	0.00137	0.71422	0.71190	0.71908
Precambrian, Proterozoic	Intrusive: granites and gneisses etc.	1.86–0.9 Ga	39	0.72203	0.00324	0.72185	0.71535	0.72800

**Fig 5:**
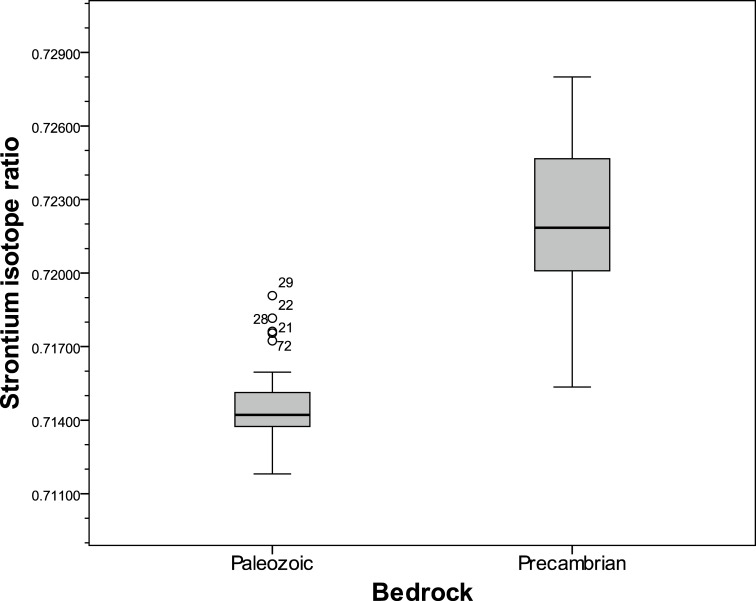
Boxplot of Sr isotope ratios in the Paleozoic and Precambrian terrains. Line: median, box: 25^th^- 75^th^ percentile, whisker: ca 95% of the data. Outliers: Sample no. 21: Hångsdala 82, no. 22: Munkestenskälla, no. 28: Hällekis säteri, no.29: St Brigidakällan, and no. 72: Borgunda.

Values in the Precambrian area have rather high standard deviation while the variation in the Lower Paleozoic areas, with sedimentary rocks, is much lower. If the outliers are considered the higher variation in the Precambrian areas is even more pronounced (Fig 5).

The difference in Sr isotope values between samples collected in the Precambrian and samples from the Paleozoic areas is highly significant (MWU-test: p < 0.001). Thus, the isotope ratios largely reflect the main divisions in bedrock geology. This is one of the main results of this study, and we can thus confirm the indications from earlier studies.

In other Paleozoic sedimentary areas, such as Östergötland, Öland and Gotland (Fig 1), baseline values similar to those in Falbygden have been reported [24, 39, 42, 43, 47, 48, 107]. However, in these regions the variation of the bioavailable Sr seems higher and in the Baltic islands Öland and Gotland Sr isotope ratios as low as 0.708/0.709 have been measured in soil and plants [24, 39, 42, 43, 47, 48, 107].

#### Variations within the Precambrian terrain

**Fig 6:**
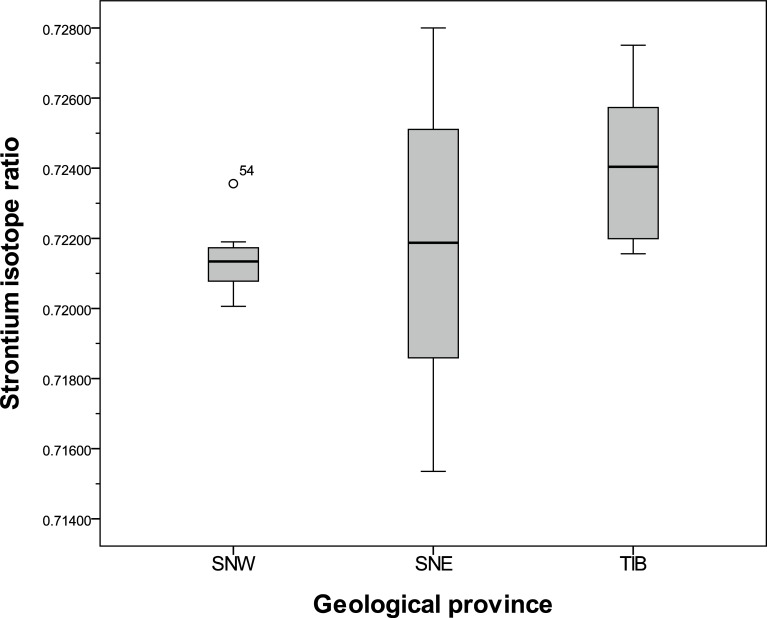
Boxplot of Sr isotope ratios from geological provinces in the Precambrian area. SNW: Sveconorwegian West, SNE: Sveconorwegian East, TIB: Transscandinavian granite-porphyry belt. Outlier: Sample 54: Råda Ås.

Table 3 shows summary statistics for samples from the different geological provinces within the Precambrian area. The means and medians from the two Sveconorwegian segments are similar, while the average and median from the easternmost province, TIB, are higher (Table 3, Fig 6). As seen in the standard deviations, the variation is considerably higher in the SNE segment than in the other regions, also when the outlier is excluded (Table 3, Fig 6).

**Table 3:** Summary statistics of Sr isotope ratios in the Precambrian area of southwestern Sweden.

Geological province/segment	Formation Age	N	Mean	Std. Deviation	Median	Min.	Max.
SNW	Early and Middle Proterozoic (1.7–0.9 Ga)	9	0.72140	0.00104	0.72134	0.72006	0.72356
SNE	Early and Middle Proterozoic (1.7–0.9 Ga)	24	0.72174	0.00380	0.72188	0.71535	0.72800
TIB	Early Proterozoic (1.8–1.6 Ga)	6	0.72415	0.00230	0.72404	0.72156	0.72751

SNW: Sveconorwegian West, SNE: Sveconorwegian East, TIB: Transscandinavian granite-porphyry belt.

Statistically, there is a significant difference between the westernmost segment (SNW) and the easternmost province (TIB), with the highest ratios measured in samples from the TIB (Tables 3 and 4). The high ratios in samples from the TIB correspond to published reference samples from the Precambrian regions of eastern Sweden [24, 46, 95, 108]. The Motala area, east of the region investigated in this study and east of Lake Vättern, on the edge of a sedimentary plain surrounded by Precambrian bedrock (Fig 1), has recently been subject to Sr isotope analyses [24]. The bioavailable baseline levels in the Cambro-Silurian sedimentary terrain range from 0.714 to 0.728 while values from the Precambrian area of the TIB and the Svecofennian province (Fig 1) range from 0.731 to 0.743, which could be expected from the older bedrock [24]. Even though these values have been measured in other sample material (largely soil samples), these results confirm higher isotope ratios of the bioavailable Sr in the more eastern and northern parts of southern Sweden, which have also been verified in other recent papers [44, 46].

**Table 4:** MWU-test of Sr isotope ratios from the geological provinces of the Precambrian area. Bold: significant at the 5% level.

	P value
TIB-SNE	0.174
TIB-SNW	**0.012**
SNE-SNW	0.766
TIB- SNE+SNW	0.063

Due to high variation the difference between SNE and the two other regions is not statistically significant, even if the outlier (sample 54) is removed. However, the ^87^Sr/^86^Sr values lay between the range of values of the two other regions, and thus agree with their geographical position (Fig 1). Hence, the results seem to reflect the general differences in formation ages of the three terrains.

**Fig 7:**
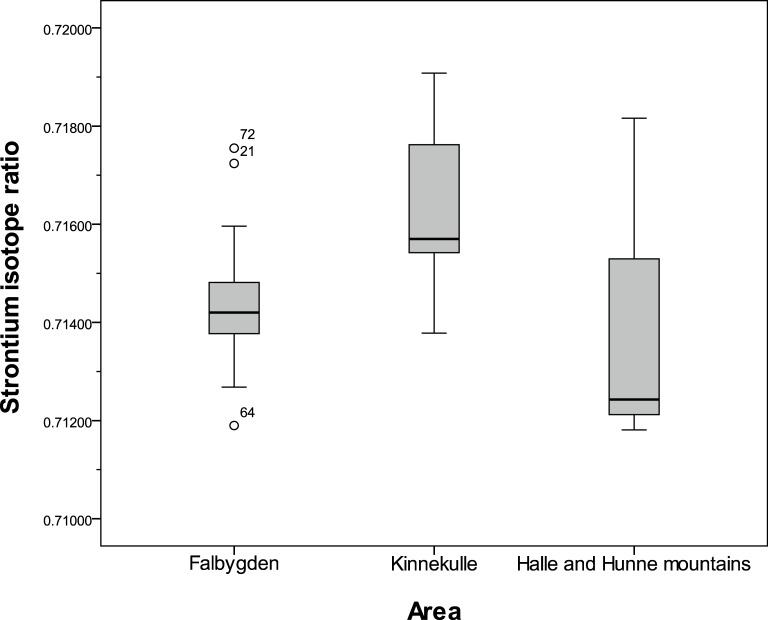
Boxplot of Sr isotope ratios in the sedimentary areas of southwestern Sweden. Outliers: Sample no. 21: Hångsdala 82, no. 72: Borgunda and no. 64: Falköping stad 3.

Both the lowest and highest ratios in the Precambrian area are found in the SNE segment (Fig 7). The lowest (No. 78: ^87^Sr/^86^Sr = 0.7154) derives from a pike collected at a medieval site 30 km south of Falbygden and the highest (No. 37: ^87^Sr/^86^Sr = 0.7280) comes from spring water sampled on pasture land 34 km southwest of Falbygden (S1 Appendix). The high variation of ^87^Sr/^86^Sr values from the SNE can partly be explained by the occurrence of intrusive rocks in some areas (Figs 2 and 3).

Samples from sites south of Falbygden (no. 45, 55, 75, 78) show relatively low Sr isotope ratios, which partly could be explained by the occurrence of gabbros in this area (Fig 2). Another factor is admixture of Sr from ice transported sedimentary rocks from Falbygden as proposed by Gillberg [101], Frei [40] and Sjögren, Price and Ahlström [8].

The presence of gabbro in a narrow band south of Lake Vänern (Fig 2) could explain the lower Sr isotope ratio (0.7157) in sample No. 43 in this area. Unfortunately, this sample is rather isolated and further sampling in this area would be recommended in the future. The high Sr isotope ratio (0.7236) in sample no. 54 collected at the Råda Ås spring (outlier in Fig 6) could possibly be explained by “lake spray” from Lake Vänern which is characterized by a similarly elevated Sr isotope value (0.7244). However, the high ratio could also be a result of admixture of Sr from ice transported Precambrian material stemming from areas to the northeast. The high ^87^Sr/^86^Sr ratio of sample no. 37 is more difficult to explain and considering the geological background such elevated signals would be more expected in samples from the TIB, exposed to the east and north of Falbygden. Again, this sample might be influenced by more non-local contributions of Sr from long distance ice transported material from the northeast.

Overall, the Sr isotope ratios are consistent with the expected values of the underlying bedrocks of the respective area, but on a more local scale, some deviations can be discerned. As shown in Fig 2, the Precambrian basement rocks have a complex areal distribution with several different geological subunits. These subunits are geologically and chronologically discernible and consist of certain distinctive rock types as categorized by the Swedish Geological Survey (Fig A and Table D in S2 Appendix). A more detailed investigation of the Sr isotope ratios measured in samples from these different subunits is available in S2 Appendix. There is a statistically significant difference only between the Sr isotope values from samples collected in the two subunits 546 and 547 (Fig 2), who dominate the SNE segment. The difference between these two subunits consists in higher ^87^Sr/^86^Sr values in subunit 546, mostly present in the northern and eastern part of the SNE segment (S2 Appendix, Table D in S2 Appendix).

#### Paleozoic areas

In the Paleozoic areas, Cambro-Silurian sedimentary rocks are partly capped by Devonian-Cambrian diabase sills. As shown earlier, the bioavailable Sr isotope signals measured in samples from the Paleozoic areas are significantly lower than the signals from the Precambrian areas.

The samples taken from the different sedimentary substrates are quite similar and have low variability, although values on sandstone have a somewhat higher mean and median (Table 5). The sandstone is of a different origin and contains older material than the other sedimentary rocks. Statistical testing shows that none of the differences were significant (S2 Appendix). The covering moraine and other soils have probably evened out the variances in Sr isotope ratios of the underlying geology.

**Table 5:** Summary statistics of Sr isotope ratios from localities in Paleozoic areas.

Area	Lithology	N	Mean	Std. Dev.	Median	Min.	Max.
Halle and Hunne mountains	All	3	0.71413	0.00350	0.71243	0.71181	0.71816
	Diabase	2	0.71212	0.00044	0.71212	0.71181	0.71243
	Sandstone	1	0.71816				
Kinnekulle	All	5	0.71632	0.00206	0.71570	0.71378	0.71908
	Slate	1	0.71570				
	Limestone	1	0.71542				
	Alum shale	2	0.71570	0.00272	0.71570	0.71378	0.71762
	Sandstone	1	0.71908				
Falbygden	All	40	0.71440	0.00106	0.71420	0.71190	0.71755
	Diabase	1	0.71724				
	Slate	5	0.71409	0.00091	0.71405	0.71310	0.71554
	Limestone	19	0.71449	0.00098	0.71421	0.71330	0.71755
	Alum shale	10	0.71401	0.00117	0.71417	0.71190	0.71558
	Sandstone	5	0.71459	0.00046	0.71459	0.71414	0.71527

The diabase is the youngest bedrock found within the study area and we would expect bioavailable Sr derived from these rocks to show the least radiogenic Sr isotope signals. However, the samples from diabase are too few for statistical evaluation, and are also very divergent. In the Halle and Hunne region, water samples from lakes on top of the diabase returned the lowest ^87^Sr/^86^Sr ratios measured in water (Table 5, S1 Appendix). Contrary to this, the single water sample collected from a location on diabase in Falbygden (discussed below), revealed the highest Sr ratio (0.7172) measured in this area (Table 5, S1 Appendix).

We also compared the three sedimentary areas of Falbygden, Kinnekulle and the Halle and Hunne mountains including the samples on diabase (Fig 7, Tables 5 and 6). There are significantly higher values at Kinnekulle than in Falbygden while other differences are not significant. The higher values at Kinnekulle could be explained by a smaller contribution of bioavailable Sr derived from diabase and a higher contribution from sandstone, but more probably by larger amounts of ice transported Precambrian material. From Halle and Hunne mountains and Kinnekulle only eight samples were collected (Table 5) and even though the areas are rather small, further samples would be desired. In Falbygden, the range of ^87^Sr/^86^Sr values is narrower than that of the other two sedimentary areas, despite a few outlier values which are discussed below (Fig 7).

**Table 6:** MWU-test of Sr isotope ratios in the three sedimentary areas. Bold: p<0.05.

	Falbygden	Kinnekulle
Halle and Hunne mountains	P = 0.256	P = 0.393
Falbygden		**P = 0.026**

The isotope ratios measured in the sandstone areas in Falbygden are slightly lower than the ratios from Halle and Hunne mountains and at Kinnekulle (Table 5). This is probably a result of less radiogenic Sr derived from thicker layers of soils, with primarily Cambro-Silurian material, covering most of the sandstone in Falbygden [97, 102].

#### Falbygden

**Fig 8:**
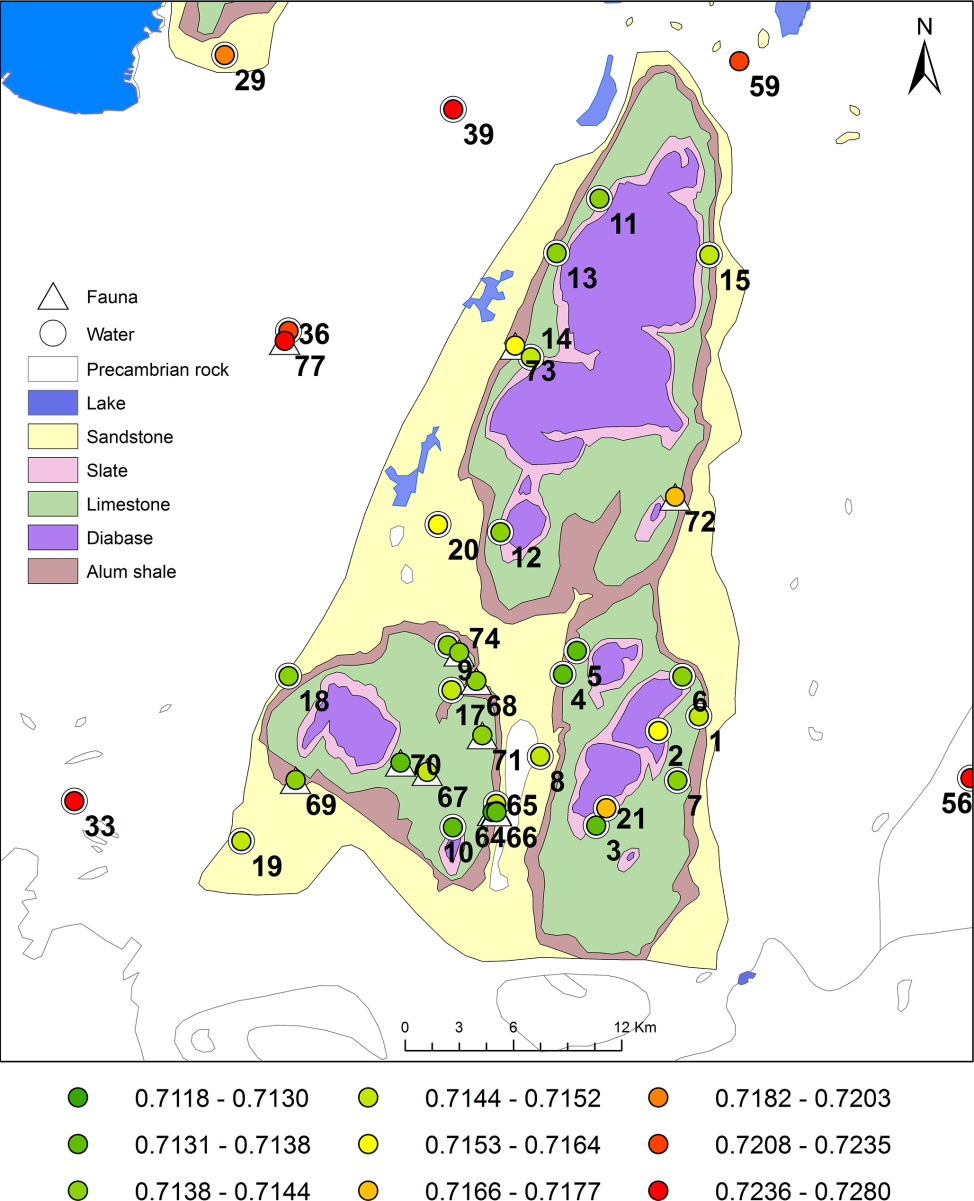
Geological map of Falbygden with sample locations and Sr isotope ratios, by Malou Blank (see S1 Appendix). Geological data from the Geological Survey of Sweden. Background map created using data from Esri. The isotopic ranges are classed by geometrical intervals in 9 different groups. A: Mösse- and Ålleberg, B: Gerums- and Varvsberget and C: Billingen.

In Falbygden, were most of the samples were collected, the spatial resolution is high with 21 water and 19 animal samples (Fig 8).

The mean and median ^87^Sr/^86^Sr values of different rock types are very similar (values around 0.714 but slightly higher, 0.715 on sandstone) except for the sample on diabase showing a much higher ratio (^87^Sr/^86^Sr = 0.7172). The variation of ^87^Sr/^86^Sr values is low (Table 5, Fig 7). Statistical significance tests revealed no significant difference between samples from any of the rock types (Tables 5 and 7).

**Table 7:** MWU-test of Sr isotope ratios from different sedimentary substrates in Falbygden.

	Limestone	Alum shale	Sandstone
Slate	P = 0.297	P = 0.859	P = 0.222
Limestone		P = 0.429	P = 0.489
Alum shale			P = 0.371

The low variation of Sr isotope ratios within Falbygden is probably a result of efficient averaging of various components (Paleozoic sedimentary rocks, Precambrian material and Devonian diabase) by glacial processes and the deposition of these thoroughly mixed tills on top of the Cambro-Silurian layers. Local variation is still possible but must then be on a much smaller spatial scale, not coinciding with bedrock type. There are a few exceptions with deviating ^87^Sr/^86^Sr values, defined as outliers in Figs 5 and 7 and Fig C in S2 Appendix. Sample no. 21 from a small lake on diabase [40, 41] exhibits a rather high Sr isotope ratio, 0.7172. This was sampled in 2009 and the circumstances of the sample are not clear [41] and should therefore be considered with caution. A plausible explanation for this high ratio is a local deposit of Precambrian material, and this sample is likely not representative for values on diabase. This might also suggest that Sr derived from weathering diabase is not an important component of the overall bioavailable Sr fraction in this area. The lowest ratio measured in Falbygden, ^87^Sr/^86^Sr 0.7119, derives from a snail shell (sample 64) which has been discussed as a species showing ^87^Sr/^86^Sr values that are biased towards calcareous components of the soil or rainwater (see above). Sample no. 72 is sampled on a rodent from the eastern part of Falbygden and the high ^87^Sr/^86^Sr ratio could indicate that this animal originates from the outskirts of Falbygden or even further away, or it might be a modern rodent affected by non-local sources. However, we cannot exclude the possibility that this animal is affected by isolated pockets of Precambrian glacial deposits.

The glacial movements could have affected areas of Falbygden differently, in terms of various types and amount of ice transported material, as suggested by Sjögren, Price and Ahlström [8]. To test this theory, we investigated if potential Sr isotope variations can be correlated with geographical location within Falbygden. For this purpose the samples were divided into three geographical areas (Fig 8): a southwestern part around Mösse- and Ålleberg (A), a southeastern part around Gerums- and Varvsberget (B) and the northern Billingen area (C).

As seen in Tables 8 and 9, there are no significant differences in ^87^Sr/^86^Sr values at the 5% level between the three areas, although there is a significant difference at the 10% level with higher ratios in the northern area of Falbygden. In Fig 8, a pattern of samples with higher ratios along the eastern and western borders of Falbygden might also be discerned.

**Table 8:** Summary statistics of Sr isotope signals in different parts of Falbygden.

Area	N	Mean	Std. Dev.	Median	Min.	Max.
Southwest (A)	23	0.71415	0.00089	0.71418	0.71190	0.71596
Southeast (B)	9	0.71450	0.00125	0.71418	0.71327	0.71724
North (C)	8	0.71502	0.00116	0.71476	0.71405	0.71755

**Table 9:** MWU-test of Sr isotope ratios in different parts of Falbygden.

	Southeast (B)	North (C)
Southwest (A)	P = 0.837	P = 0.074
Southeast (B)		P = 0.321

### Surface model

**Fig 9:**
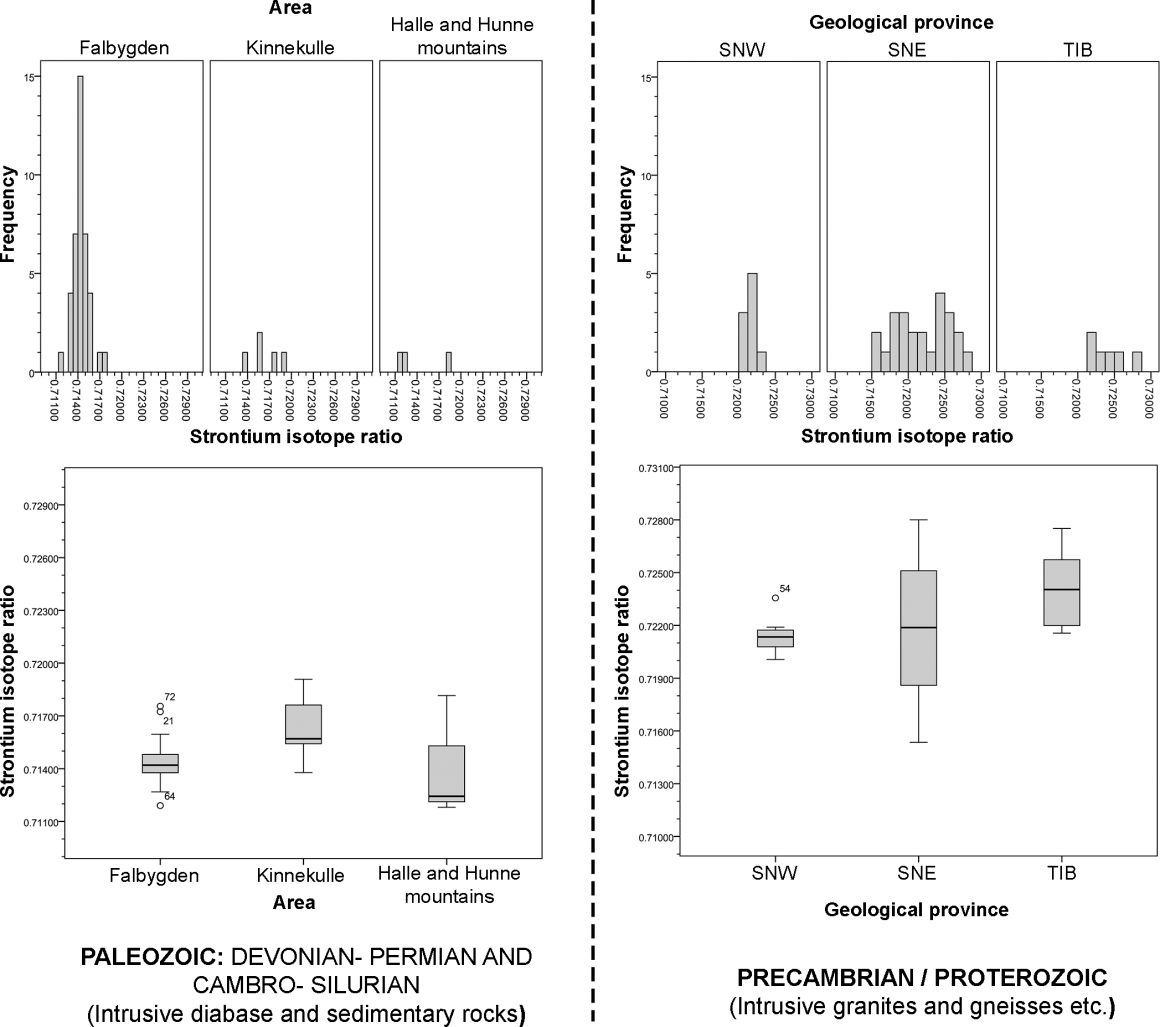
Overview of Sr isotopes ratios of water and animal samples from different Palaeozoic and Proterozoic provinces presented by boxplots and histograms.

When comparing our results from different geological substrates, several areas with distinct baseline ranges appear. Fig 9 clarifies both the differences and the overlaps of the bioavailable Sr isotope values in the study area. The outliers are considered to represent either non-local fauna or very localized anomalies. In any case, these outliers are unlikely to have a significant impact on the overall bioavailable Sr signature of a common human home range.

**Fig 10:**
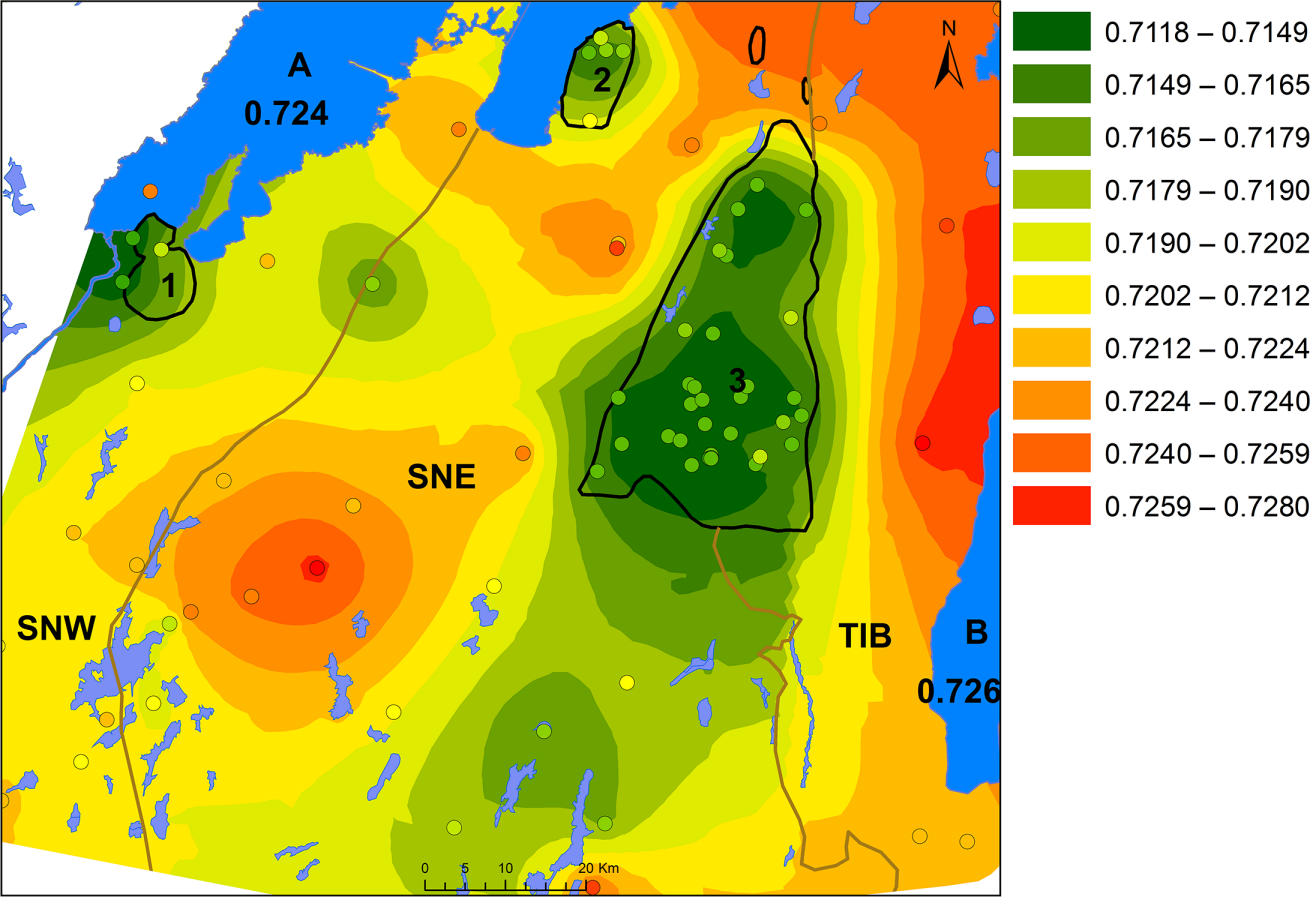
An interpolated surface of the Sr isotope ratios of water and animal samples, by Malou Blank and Karl-Göran Sjögren. Background map created using data from Esri. The ranges are classified by natural breaks in 10 groups. 1: Halle and Hunne mountains, 2: Kinnekulle, 3: Falbygden. A: Lake Vänern, B: Lake Vättern. SNW: Sveconorwegian west, SNE: Sveconorwegian east and TIB: Transscandinavian granite-porphyry belt.

The wide range of Sr isotope ratios in the Precambrian terrain, the overlap of Sr isotope ratios between different geological areas as well as the overlay of sediments and moraine is not ideal for modelling the Sr isotope distribution according to bedrock type. Therefore, an Empirical Bayesian kriging function offered by ArcGis was used to produce an interpolated surface of the Sr isotope ratios independently of bedrock or soil type (Fig 10).

**Fig 11:**
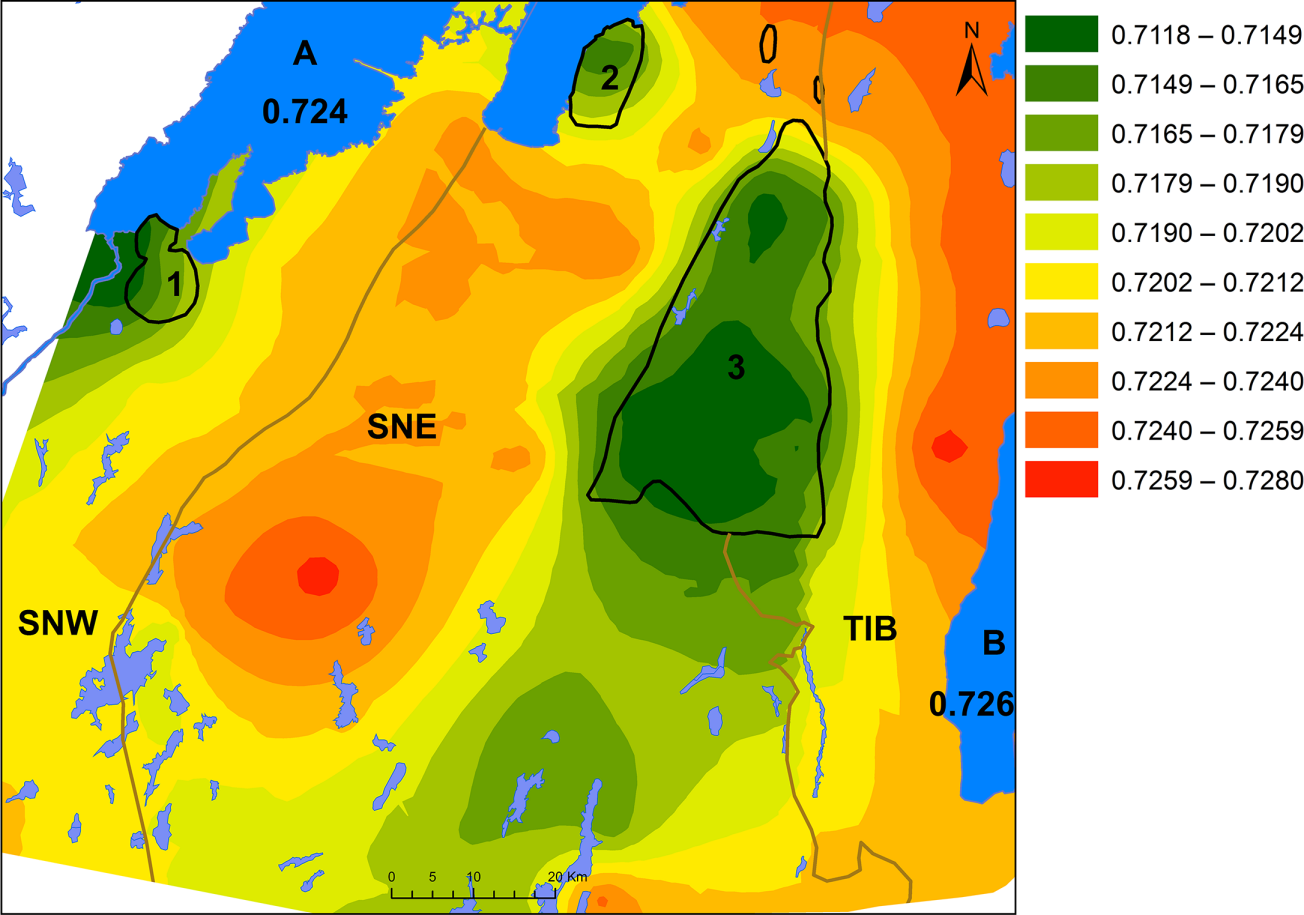
An interpolated surface of the Sr isotope ratios of water and animal samples from southwestern Sweden, by Malou Blank. Sample 43 excluded. Background map created using data from Esri. The ranges are classified by natural breaks in 10 groups. 1: Halle and Hunne mountains, 2: Kinnekulle, 3: Falbygden. A: Lake Vänern, B: Lake Vättern. SNW: Sveconorwegian west, SNE: Sveconorwegian east and TIB: Transscandinavian granite-porphyry belt.

The interpolated surface is a generalisation of the individually measured isotope ratios of the samples, predicting the isotope composition of bioavailable Sr in the intervening areas. The highest ^87^Sr/^86^Sr values are, as expected, concentrated in the eastern parts and the lowest values agree with those measured on samples from the younger sedimentary rocks, such as Falbygden (Figs 10 and 11). The model also reveals that low ^87^Sr/^86^Sr values are to be found in an area extending towards the south-southwest of Falbygden. A similar pattern can be observed with high limestone and calcium in the moraines south of Falbygden [101, 103]. This trend is compatible with the general direction of movement of the glacial ice in the area [102]. Additional samples from this area are necessary to further substantiate this trend.

The interpolation model does not take bedrock composition into consideration, which might lead to an unrealistic smoothing over some bedrock borders in some cases, such as between the younger sedimentary areas and the older intrusive rock terrain. Also, the variable sampling densities are problematic for most interpolation methods, although less so for kriging. The outliers discussed above (Fig 9), do not have a large impact on the model except if they are isolated samples. The model would benefit from additional samples in parts of the Precambrian area. As an example, a critical area between the SNW and SNE segments is less well defined due to very sparse sampling (Fig 10). Here, sample 43, with a very low ^87^Sr/^86^Sr ratio discussed earlier, seems to have too much impact on the interpolated surface as the lack of close neighbouring samples causes this sample to appear as an isolated low ratio island (Fig 10). The effect of removing this particular sample from the model is shown in Fig 11. Even though there are some weaknesses, we find that the model is still a good prediction of expected bioavailable Sr isotopic ratios in southwestern Sweden.

## Conclusion

In this study we present the first extensive Sr isotope baseline in Sweden, covering an area of 120 x 130 kilometres of the southwestern inland. Several areas with different baseline ranges can be distinguished, although with overlaps between some areas. Questions considering the construction of baselines were addressed and insights into different proxies highlighted. In our case statistical analyses showed that small non-domestic mammals and water samples were compatible.

Our Sr isotope measurements of water and fauna samples largely reflect the underlying bedrock geology of the area. The highest Sr isotope ratios are found in samples located in the oldest geological provinces, while the lowest ratios where measured in samples from the areas comprising the youngest bedrock in the study area. We demonstrate that there is a significant difference between the isotope ratios of the bioavailable Sr of the Paleozoic sedimentary and intrusive rock terrains and the Precambrian granitoid-gneiss areas. Glacially transported till and other glaciogenic materials are a likely cause for deviations from this pattern, increasing the ^87^Sr/^86^Sr signals from the younger sedimentary areas and lowering the ^87^Sr/^86^Sr isotope composition of samples in parts of the older Precambrian terrains.

The isotope ratios in samples from the Precambrian regions are internally varied. The wide range of isotope ratios in the Precambrian terrain can be potentially explained by the complex geology with metamorphic events and glacial deposits, which characterizes the area. Based on our statistical evaluations, there is a significant difference between ^87^Sr/^86^Sr values of samples in the most western segment (SNW) and the easternmost province (TIB), with the highest ratios measured in samples from the TIB. In the Precambrian area south of Falbygden, there are sites with lower ratios than expected, probably due to soil deposits by glaciers and the presence of younger intrusive rocks.

Samples from the Paleozoic sedimentary areas are characterized by significantly lower and less variable Sr isotope signatures than the Precambrian areas. The lowest variation of Sr isotope ratios was measured in Falbygden, where most of the samples were collected. The narrow Sr isotope range of these sedimentary areas can tentatively be explained by effective mixing and averaging out of various components, such as Paleozoic sedimentary rocks, Precambrian material and Devonian diabase by glaciogenic processes and the deposition of these thoroughly mixed tills on top of the Cambro-Silurian bedrock. The lowest isotope ratios were measured in samples from locations dominated by diabase, the youngest intrusive rock in the area.

Furthermore, ^87^Sr/^86^Sr values measured in samples from the sedimentary area of Falbygden are significantly lower than in samples from the sedimentary area of Kinnekulle. A possible explanation is differences in the proportions of sedimentary rocks, diabase and Precambrian moraine deposits.

We also produced interpolated surface models to predict the isotopic ranges of the bioavailable Sr in the area. This model corresponds well with the other results and shows the influence of the glacial movements in the region, in addition to bedrock substrate. It also shows the narrow Sr isotope range of Falbygden and the subregions with differing Sr isotope ratios within the Precambrian province.

Our background data allows for more nuanced and detailed interpretations of human and animal mobility in the region, and we can now identify long distance mobility with greater confidence, eg. from eastern Sweden.

It is our hope that this study will be a basis for future research that compiles more samples and achieves a more detailed resolution.

## Supporting information

S1 Appendix Sample information, Excel file. (XLSX)

S2 Appendix Supplementary information: Geological supplementary information, A comparison of ^87^Sr/^86^Sr values in fauna and water samples, Strontium isotope ratios in samples from different Precambrian subunits, Strontium isotope ratios in samples from different sedimentary lithologies. (DOCX)
