# Supplementary material for: Isotope values of the bioavailable strontium in inland southwestern Sweden—A baseline for mobility studies
Source: PLoS One. 2018 Oct 4;13(10):e0204649. doi: 10.1371/journal.pone.0204649 (PMC6171848; doi:10.1371/journal.pone.0204649)
Supplement: S2 Appendix — (DOCX) [file pone.0204649.s002.docx]

# S2 Appendix

**Supplementary information**

**Geological supplementary information**

**
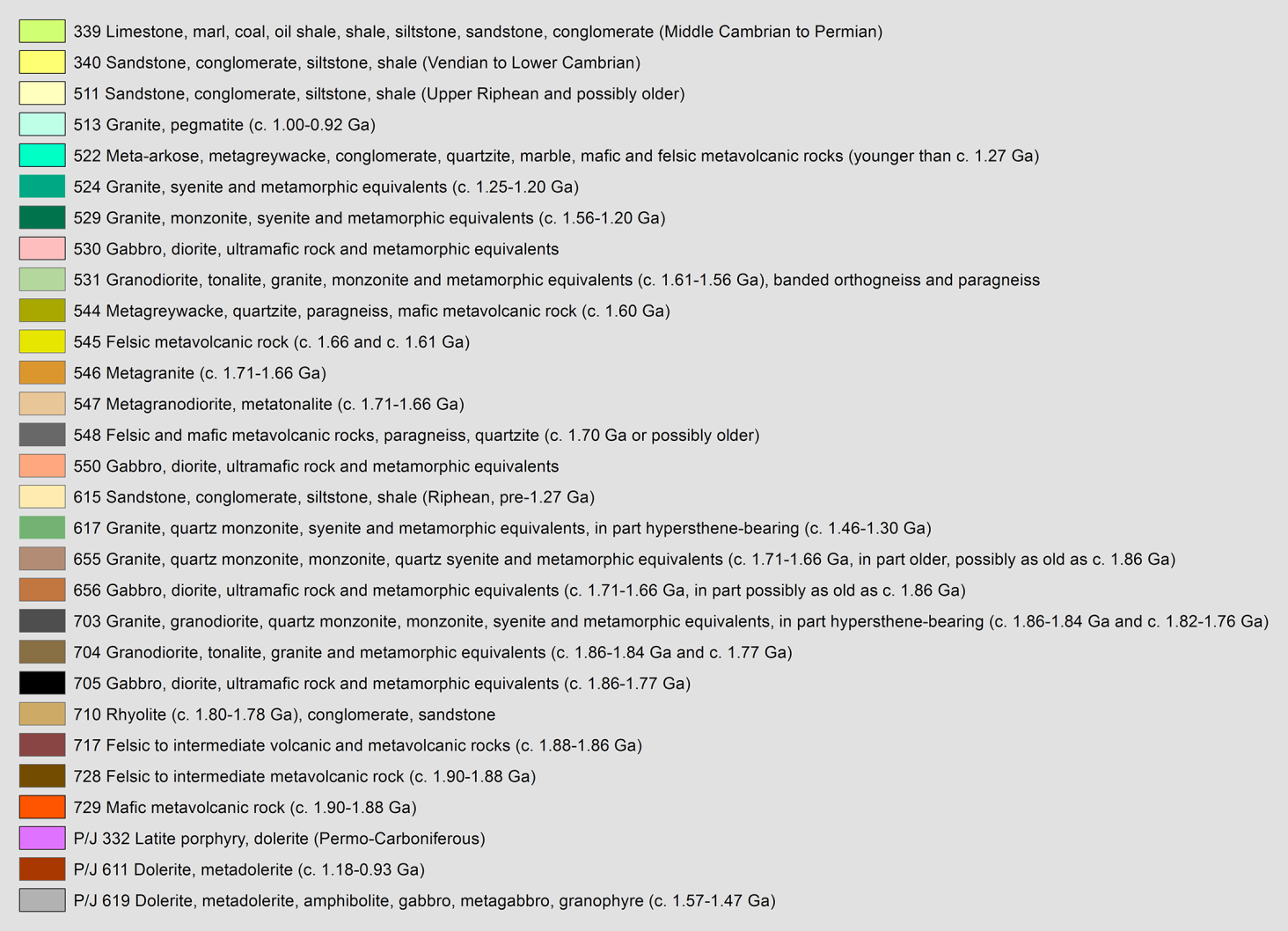
**

**Fig A. Summary of the various rock types in the different geological subunits as defined by the Swedish Geological Survey.**

**
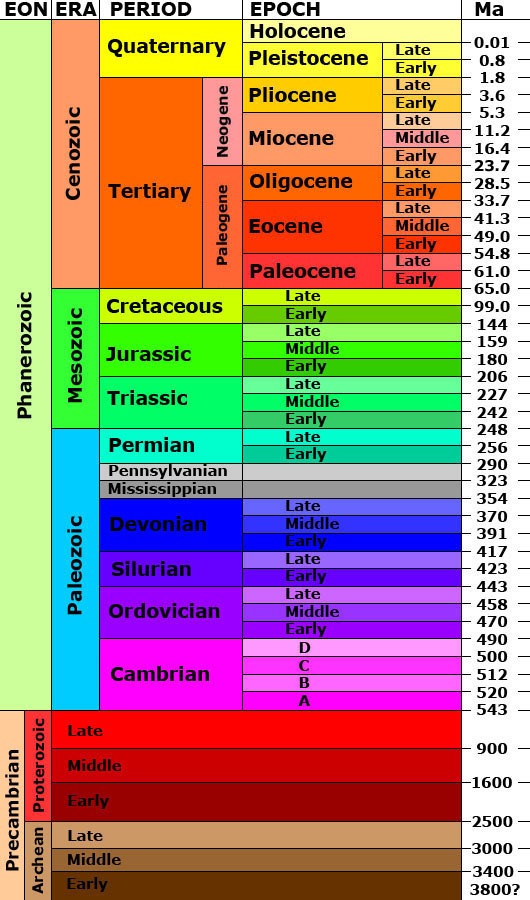
**

**Fig B. Overview of the geological periods.**

**A comparison of ^87^Sr/^86^Sr** **values in fauna and water samples**

A comparison of the different sample types was conducted. The various types of water sources were contrasted and thereafter fauna and water samples from Falbygden were compared. As only a few faunal samples from the Precambrian area were available and these were not collected in the vicinity of any water samples, these were not considered suitable for this investigation (Fig 2, S1 Appendix). In Falbygden, on the other hand, the number of reference samples is relatively high with 19 fauna and 21 water samples.

The water samples from Falbygden were divided into two categories: springs and creeks/streams, with respect to how much they were influenced by groundwater sources (see previous discussion). Thirteen spring samples were compared with seven creek/stream/lake samples (S1 Appendix). One sample of pore water (no. 21) of uncertain origin was not included in this test. The mean values, medians and standard deviations are very similar in the samples more affected by the surface water (i.e. creek/streams samples) and the samples more affected by groundwater (eg. springs) (Table A), and no significant difference could be observed between the two categories (MWU-test: p=0.699). Hence, in our study area we see no complications in combining the two different water sample categories.

**Table A. Summary statistics of strontium isotope ratios in Falbygden water samples.**

| Sample type | | N | Mean | Std. Dev. | Median | Min. | Max. |
| --- | --- | --- | --- | --- | --- | --- | --- |
| Water | Creek/stream/lake | 7 | 0.71422 | 0.00066 | 0.71418 | 0.71327 | 0.71527 |
|  | Spring | 13 | 0.71432 | 0.00067 | 0.71421 | 0.71310 | 0.71554 |
|  | Total | 20 | 0.71428 | 0.00090 | 0.71420 | 0.71310 | 0.71554 |

We then compared fauna and water samples from Falbygden and from lithologies where both sample types are available (Tables B and C). These values were not statistically significantly different (Tables B and C), which is also illustrated in the boxplots where medians and outliers are shown for all samples (Fig C). We note that faunal samples are more heterogeneous in their ^87^Sr/^86^Sr values. However, since statistically not discernible from each other, we consider the fauna and water samples to be compatible and they will therefore be lumped in the following.

**Table B. Summary statistics of strontium isotope ratios in Falbygden water and fauna samples.**

| Sample type | Lithology | N | Mean | Std. Dev. | Median | Min. | Max. |
| --- | --- | --- | --- | --- | --- | --- | --- |
| Water | Limestone | 10 | 0.71432 | 0.00048 | 0.71435 | 0.71330 | 0.71491 |
| Fauna | Limestone | 9 | 0.71467 | 0.00135 | 0.71418 | 0.71347 | 0.71755 |
| Water | Alum shale | 1 | 0.71327 |  | 0.71327 | 0.71327 | 0.71327 |
| Fauna | Alum shale | 9 | 0.71410 | 0.00121 | 0.71422 | 0.71190 | 0.71558 |
| Water | Sandstone | 4 | 0.71470 | 0.00047 | 0.71468 | 0.71414 | 0.71527 |
| Fauna | Sandstone | 1 | 0.71419 |  | 0.71419 | 0.71419 | 0.71419 |

**Table C. MWU-test on strontium isotope ratios of fauna and water samples from Falbygden.**

|  | P value |
| --- | --- |
| Limestone | 1.000 |
| Alum shale | 0.600 |
| Sandstone | 0.800 |
| All samples | 0.915 |

**
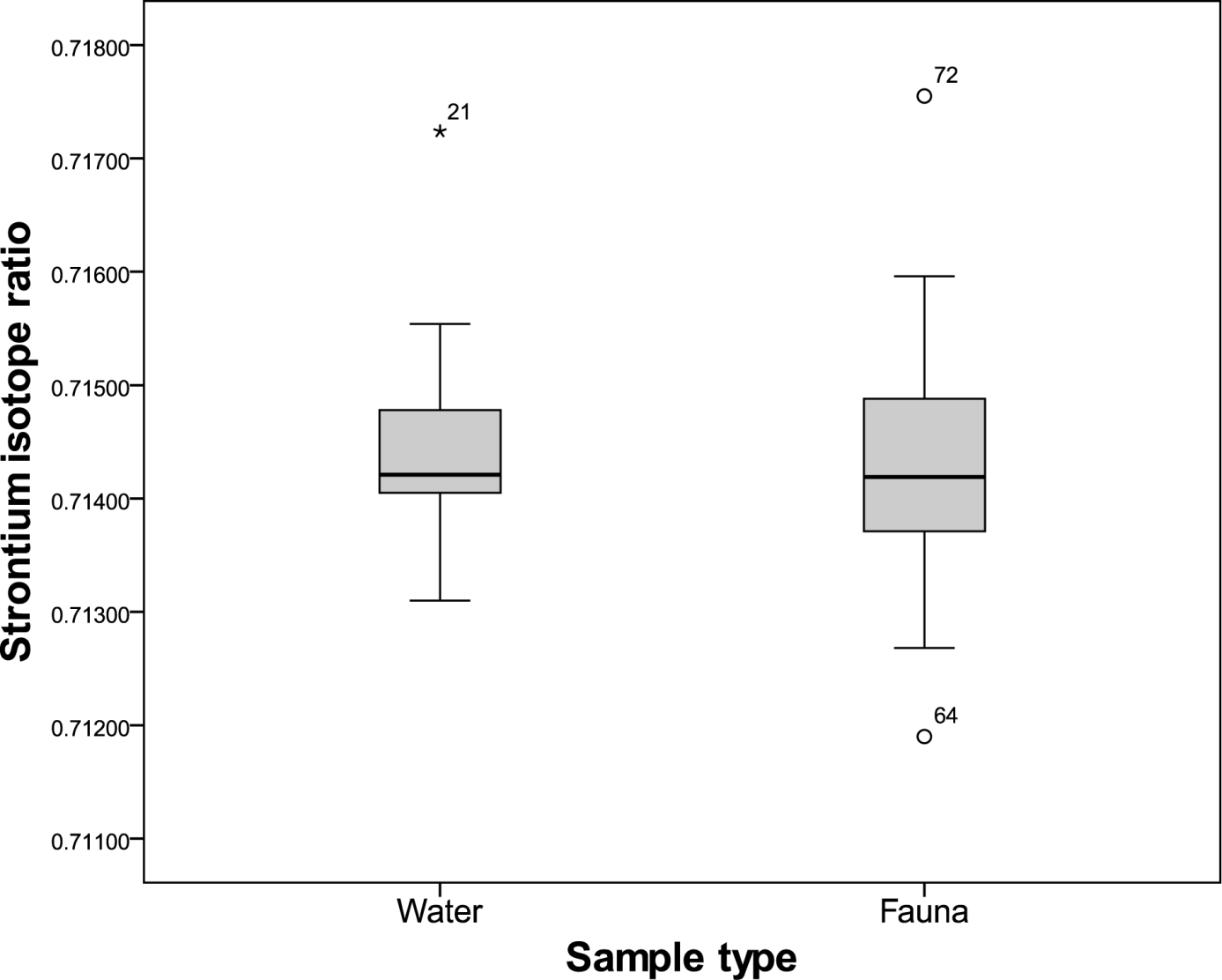
**

**Fig C. Boxplot of strontium isotope ratios of all water and fauna samples in Falbygden**. Line: median, box: 25^th^- 75^th^ percentile, whisker: ca 95% of the data. Outliers: Sample no. 21: Hångsdala 82, no. 72: Borgunda and no. 64: Falköping stad 3.

**Strontium isotope ratios in samples from different Precambrian subunits**

In this part the strontium isotope ratios of samples collected in the different subunits are discussed. In Table D summary statistics of these subunits are presented.

**Table D. Summary statistics of strontium isotope ratios in Precambrian subunits.**

| Province | Subunit | Formation Age | N | Mean | Std. Dev. | Median | Min. | Max. |
| --- | --- | --- | --- | --- | --- | --- | --- | --- |
| SNW | 530: Gabbro, diorite, ultramafic rock and metamorphic equivalents | Middle Proterozoic (1.5- 1.3 Ga) | 1 | 0.72356 |  | 0.72356 | 0.72356 | 0.72356 |
| SNW | 531: Granodiorite, tonalite, granite, monzonite and metamorphic equivalents, banded orthogneiss and paragneiss | Early Proterozoic (1.61- 1.56 Ga) | 5 | 0.72114 | 0.00074 | 0.72127 | 0.72006 | 0.72190 |
| SNW | 545: Felsic metavolcanic rock | Early Proterozoic (1.66- 1.61 Ga) | 3 | 0.72110 | 0.00077 | 0.72134 | 0.72024 | 0.72173 |
| SNE | 550: Gabbro, diorite, ultramafic rock and metamorphic equivalents | Middle and Late Proterozoic (1.3- 0.5 Ga) | 1 | 0.71761 |  | 0.71761 | 0.71761 | 0.71761 |
| SNE | 547: Meta-granodiorite, metatonalite | Early Proterozoic (1.71- 1.66 Ga) | 10 | 0.72005 | 0.00284 | 0.71934 | 0.71535 | 0.72460 |
| SNE | 546: Metagranite | Early Proterozoic (1.71- 1.66 Ga) | 13 | 0.72335 | 0.00387 | 0.72473 | 0.71569 | 0.72800 |
| TIB | 655: Granite, quartz monzonite, monzonite, quartz syenite and metamorphic equivalents | Early Proterozoic (1.86- 1.66 Ga) | 4 | 0.72451 | 0.00264 | 0.72448 | 0.72156 | 0.72751 |
| TIB | 703: Granite, granodiorite, quartz monzonite, monzonite, syenite, in part hypersthene-bearing | Early Proterozoic (1.86- 1.76 Ga) | 2 | 0.72342 | 0.00202 | 0.72342 | 0.72199 | 0.72485 |

SNW: Sveconorwegian West, SNE: Sveconorwegian East, TIB: Transscandinavian granite-porphyry belt. The subunit numbers refer to geological zones plotted in Figure 2, and explained in Supplement 2.

The single sample (45) from the Precambrian subunit 550, in the SNE province, has much lower, while the sample (54) from subunit 530, in the SNW province, has much higher ^87^Sr/^86^Sr ratios than the other units of similar age (Fig 3). The small number of samples from most subunits hinders further statistical investigation, with the exception of subunits 546 and 547 which cover most of the SNE province and where a large number of locations were sampled (Fig 2, Table D). Between these two subunits there is a statistically significant difference in strontium isotope ratios (MWU-test, p=0.021), with higher values in 546 which is mostly present in the northern and eastern part of the SNE province (Fig 2, Table D). The differences of ^87^Sr/^86^Sr values of samples from these two subunits can be explained by the nature of the basement: subunit 546 is dominated by metagranites, where in subunit 547 less differentiated metatonalites and metagranodiorites prevail. Generally, granites have higher Rb/Sr ratios than granodiorites and tonalities, which impart, over geological time, higher ^87^Sr/^86^Sr ratios in the former rocks compared to the latter. In addition, the thicker layer of marine and lake sediment packages in the northern part, probably contribute with an extra radiogenic strontium component to the bioavailable strontium in this northerly part. Deposits of soil material from the east might also have affected the northern and eastern part of this province and, thus, resulting in higher strontium isotope ratios.

**Strontium isotope ratios in samples from different sedimentary lithologies**

Statistical analyses were conducted based on the different sedimentary lithologies (Tables E and F). The samples taken from the different sedimentary substrates are quite similar and have low variability, although values on sandstone have a somewhat higher mean and median.

**Table E. Summary statistics of strontium isotope ratios of water and animal samples from localities on Paleozoic sedimentary rocks.**

| Lithology | N | Mean | Std. Dev. | Median | Min. | Max. |
| --- | --- | --- | --- | --- | --- | --- |
| Slate | 6 | 0.71436 | 0.00105 | 0.71411 | 0.71310 | 0.71570 |
| Limestone | 20 | 0.71453 | 0.00097 | 0.71435 | 0.71330 | 0.71755 |
| Alum shale | 12 | 0.71430 | 0.00149 | 0.71417 | 0.71190 | 0.71762 |
| Sandstone | 7 | 0.71574 | 0.00202 | 0.71478 | 0.71414 | 0.71908 |

**Table F. MWU-test on strontium isotope ratios of water and animal from localities on Paleozoic sedimentary rocks.**

|  | Slate | Limestone | Alum shale | Sandstone |
| --- | --- | --- | --- | --- |
| Slate |  | P=0.614 | P=0.964 | P=0.181 |
| Limestone |  |  | P=0.526 | P=0.130 |
| Alum shale |  |  |  | P=0.100 |

There are no significant differences between samples from areas with different Paleozoic rock types (Table F). There is a tendency of higher strontium isotope values at localities on sandstone than the values from the remaining sedimentary rocks. This difference is significant on a 10% level, but not on a 5% level (MWU-test, p=0.071). The Sandstone is of a different origin and contains older material than the other sedimentary rocks. The covering soils have probably evened out the variances in strontium isotope ratios of the underlying geology (see above).
